# Does Larval Rearing Diet Lead to Premating Isolation in *Spodoptera litura* (Fabricius) (Lepidoptera: Noctuidae)?

**DOI:** 10.3390/insects12030203

**Published:** 2021-02-27

**Authors:** Xue-Yuan Di, Bin Yan, Cheng-Xu Wu, Xiao-Fei Yu, Jian-Feng Liu, Mao-Fa Yang

**Affiliations:** 1 Institute of Entomology, Guizhou University; Guizhou Provincial Key Laboratory for Agricultural Pest Management of the Mountainous Region; Scientific Observing and Experimental Station of Crop Pest in Guiyang, Ministry of Agriculture, Guiyang 550025, China; xiaomdd@126.com (X.-Y.D.); 15285171818@163.com (B.Y.); 2 College of Forestry, Guizhou University, Guiyang 550025, China; muzixuan58@126.com; 3 College of Tobacco Science, Guizhou University, Guiyang 550025, China; anjingfly2009@163.com

**Keywords:** diet, mate choice, assortative mating, *Spodoptera litura*, life table

## Abstract

**Simple Summary:**

*Spodoptera litura* Fabricius (Lepidoptera: Noctuidae) is a serious polyphagous pest. Most studies focus on the effects of natural hosts on *S. litura*. However, progressively more laboratory studies *S. litura* involve feeding the larvae with an artificial diet. We compared the life performance and observed mating choice of *S. litura* reared on tobacco, Chinese cabbage, and an artificial diet. The results revealed that diet had a significant effect on the duration of each stage of development. In the multiple-choice test with individual males consuming tobacco, Chinese cabbage, or an artificial diet, females fed on the artificial diet preferred to mate with males that were fed on the same diet and rarely mated with males fed on tobacco or Chinese cabbage. We suggest that the diet of *S. litura* has a potential impact on mate choice and sexual isolation.

**Abstract:**

Host plant preference during the larval stage may help shape not only phenotypic plasticity but also behavioral isolation. We assessed the effects of diet on population parameters and mate choice in *Spodoptera litura*. We raised larvae fed on tobacco, Chinese cabbage, or an artificial diet, and we observed the shortest developmental time and highest fecundity in individuals fed the artificial diet. However, survival rates were higher for larvae on either of the natural diets. Population parameters including intrinsic rate of increase and finite rate of increase were significantly higher with the artificial diet, but this diet led to a lower mean generation time. Copulation duration, copulation time, and number of eggs reared significantly differed between diets. In terms of mate choice, females on the artificial diet rarely mated with males fed on a natural host. Our results support the hypothesis that different diets may promote behavioral isolation, affecting mating outcomes. Thus, findings for populations fed an artificial diet may not reflect findings for populations in the field.

## 1. Introduction

Selection for behavioral differences in insect species impacts the evolution of host plant specificity [1], and the behavioral choices of those insects reflects evolutionary adaptation [2]. Different host plants provide different levels of nutritional quality for different insect species [3], and they can have cascading effects on their life histories [4] including fitness, growth, and fecundity [5]. This phenomenon is common in polyphagous herbivores [6,7] such as cotton bollworms, which exhibit significant differences in population development among the various host plants [8].

An increasing number of studies are using insects reared on artificial diets, which can affect their life history traits [9,10]. For example, *Bactrocera cucurbitae* (Coquillett) larvae fed on an artificial diet produce significantly fewer ovarioles than individuals in natural host populations [11]. Hence, food selection may put an important constraint on fecundity [12]. In addition, mating preferences and assortative traits can lead to behavioral isolation in animals [13]. Host plant species can obviously change behavioral isolation by their impact on insect phenotypic plasticity in terms of mating signals, and shape mating preferences and sexual isolation [14]. The host plant preference of females directly impacts assortative mating [15], and mating preference contributes to premating reproductive isolation [15,16,17]. Furthermore, the mating activity of *Chilo suppressalis* Walker based on host-associated population has a genetic basis in which life history traits are influenced by genetics and host plants [3].

*Spodoptera litura* is a polyphagous pest that causes serious damage to economically important crops throughout the world. Different planting patterns have promoted the occurrence of *S. litura* populations in different host fields [18]. However, only a few studies have reported the effects of different host plants on the growth, development, and reproduction of *S. litura* [19,20,21,22]. The development of artificial diets has enabled the mass rearing of various insects in laboratories [23,24]. Today, much of the research on *S. litura* is conducted on individuals fed artificial diets [25,26,27]. Thus, it is important to investigate the effects of such diets on this species. We hypothesized that *S. litura* fed an artificial diet will show behavioral isolation due to diet-induced divergence in mating recognition signals.

The life table analysis method is a reliable tool for understanding insect survival, growth, fecundity, and population dynamics [28,29]. The age-stage two-sex life table takes into consideration the contributions of both sexes and accurate estimates of biological parameters and population fluctuations [30]. To understand the effects of different diets on *S. litura*, we applied this technique to individuals reared on the leaves of natural diets (tobacco, Chinese cabbage) or an artificial diet. This is the first study to compare the effects of natural and artificial diets on *S. litura* development and reproduction. Female discrimination plays an important role in sexual isolation and further impacts assortative mating [31]. Hence, we also tested the mate choices of females fed each diet. Our results provide additional insight into the role of diet in the evolution of sexual isolation.

## 2. Materials and Methods

### 2.1. Insect Collection and Rearing

*Spodoptera litura* were collected from tobacco fields in June 2017 from Lanba Village, Majiang County, Guizhou Province, China (26°29′54.65″ N, 107°37′49.05″ E). Larvae were brought back to the laboratory and fed Chinese cabbage and tobacco continuously for 28 generations. Individuals fed an artificial diet were purchased from Henan Jiyuan Baiyun Industry Co., Ltd, Henan, China. and were bred for 18 generations. The experiments were conducted under conditions of 27 °C (±1) with a photoperiod of 14:10 h light/dark and 60% (±5) relative humidity in a climate chamber. Eggs were maintained in 9 cm Petri dishes with moistened filter paper. Larvae were raised on Chinese cabbage, tobacco, or an artificial diet, respectively. Newly hatched larvae were reared in plastic cups (13 cm high, 17.8 cm diameter). After the third instar, larvae were reared in transparent plastic cases (20.4 × 35.2 × 12.3 cm^3^) until pupation. Fresh leaves and artificial diet were provided for larvae each day. When adults emerged, they were moved into plastic cups (13 cm high, 17.8 cm diameter) and provided with a piece of cotton soaked with 10% honey.

Chinese cabbage (*Brassica pekinensis* (Lour.)), tobacco (*Nicotiana tabacum* L.), and an artificial diet were used. The cabbage and tobacco were planted in plastic pots (28 cm in diameter, 25 cm in depth) kept in cages (200 × 200 × 250 cm^3^) covered with mesh screen (0.1 mm mesh size) to prevent pests from damaging them. No pesticides were used. Leaves from plants at 6–8 weeks of age were used in experiments.

The artificial diet [32] was prepared by boiling a mixture of 16 g agar powder and 400 mL water; adding 80 g soybean meal, 80 g bran, 32 g yeast extract, 16 g casein, and 400 mL distilled water and boiling for 30 min more; then adding 1.6 g sorbic acid and allowing the solution to cool to 60 °C. Then, 0.16 g cholesterol, 0.16 g inositol, 6.4 g vitamin C, and 0.8 g choline chloride were added, and the solution was cooled and stored at 4 °C.

### 2.2. Life Table Study

One hundred eggs used at the beginning form each diet treatment. Five egg masses produced by adults raised on each of the different diets were selected and placed on moistened filter paper in plastic Petri dishes (2 cm high, 9 cm diameter). The Petri dishes were kept in a climate chamber at 27 ± 1 °C and 60 ± 5% relative humidity under an L14: D10 h photoperiod. After hatching, 20 first-instar larvae were randomly selected from each egg mass, and each larva was housed separately with food in a 200 mL plastic vials (4 cm high, 10 cm diameter). Host leaves and the artificial diet were replaced every day until pupation. The quantity of food provided depended on the freshness of leaves and diets and larval age. The development, survival, fecundity, and longevity of *S. litura* fed each diet were recorded. Individual larvae were examined daily for molting and survival, and their instar stage was recorded. At adult emergence, 1-day-old females and males were paired in a 2000 mL plastic container and supplied with 10% honey solution as food. We collected egg masses laid by females every day until the females died. The number of eggs and survival of each individual were checked and recorded daily.

### 2.3. Mate Choice

Moth development on different host plants can display near reproductive isolation [33]. We used different mating designs to determine mate choice among the tobacco (T), Chinese cabbage (C), and artificial diet (A) populations of *S. litura*. No choice tests and choice tests were conducted to determine the mate choice of *S. litura* females among the tobacco, Chinese cabbage, and artificial diet populations.

#### 2.3.1. Non-Choice Tests

A single virgin 1-day-old female and male were randomly paired by placing them together in a 2000 mL plastic cup (13 cm high, 17 cm diameter); they were provided with 10% honey solution. The following combinations were established: both males and females reared on tobacco (T♀ × T♂), both fed Chinese cabbage (C♀ × C♂), and both fed artificial diet (A♀ × A♂). Each pair was observed every 10 min during the scotophase (from 6:00 p.m. to 8:00 a.m.) using a 7 W red lamp; we recorded copulation events, mating duration, and mating times. After they mated, we collected egg masses laid by females every day, and recorded fecundity and longevity. Each treatment had 30 replicates.

#### 2.3.2. Choice Tests

A virgin female from each diet group was paired with three unmated males (from each diet group) in a 2000 mL plastic cup (13 cm high, 17 cm diameter) and provided a 10% honey solution. Hence, the experimental groups were T♀ × (T♂ + C♂ + A♂), C♀ × (T♂ + C♂ + A♂), and A♀ × (T♂ + C♂ + A♂). Males from each experimental group were marked on the back of the pronotum using different acrylic paint colors. The different experimental groups, within which copulation took place, were observed and recorded every 10 min. The first pair to mate was observed and recorded in each trial. Each treatment had 20 replicates.

### 2.4. Statistical Analyses

#### 2.4.1. Life Table Analyses

Raw life history data were analyzed using an age-stage, two-sex life table [30,34] with the computer program TWOSEX-MSChart [35]. Paired bootstrap tests were used to compare differences in survival rate, longevity, and fecundity of *S. litura*. The population parameters assessed included the intrinsic rate of increase (*r*), the finite rate of increase (*λ*), the net reproductive rate (*R*_0_), the mean generation time (*T*), age-stage specific survival rates (*s_xj_*, where *x* is age and *j* is stage), age-specific survival rate (*l_x_*), age-specific fecundity (*m_x_*), age-stage specific fecundity (*f_x_*), adult pre-oviposition period (APOP), total pre-oviposition period (TPOP), age-specific maternity (*l_x_m_x_*), age-stage specific life expectancy (*e_xj_*), and reproductive value (*v_xj_*). The variances and standard errors of the population parameters were calculated using the program TWOSEX-MSChart with 100,000 random resamplings [35]. All graphs were plotted using Sigmaplot 14.0 (Systat Software Inc., San Jose, CA, USA).

#### 2.4.2. Population Projections

The data from the age-stage two-sex life table were used to simulate population growth using the program TIMING-MSChart [36]. An initial population of 10 eggs was used for each diet group.

#### 2.4.3. Mate Choice

All mate-choice analyses were performed in SPSS 21 (SPSS Inc., Chicago, IL, USA). The normality of the variance was tested using a normal distribution test, indicating that the date followed a normal distribution. One-way analysis of variance (ANOVA) and the Tukey post hoc tests were used to evaluate differences in mating times, fecundity, longevity of females, and males in the non-choice and choice test. Statistical significance is interpreted when the *p* value <0.05.

## 3. Results

### 3.1. Developmental Period

All diet groups were able to complete growth and development and produce offspring, but developmental time, longevity, and fecundity significantly differed by group (Table 1). The longest developmental times (females 39.78 d, males 39.87 d) were recorded for larvae fed tobacco, and the shortest (females 30.19 d, males 33.19 d) were for those reared on the artificial diet. There were obvious differences in egg, larval, pre-pupa, pupa, female, and male adult longevity when *S. litura* was fed on different diets. The mean fecundity of females fed the artificial diet was 1266 eggs, which was significantly higher than that of those fed Chinese cabbage (826). The survival rates from egg to adult females and to adult males were 32 and 30% for tobacco, 32 and 37% for Chinese cabbage, and 31 and 31% for artificial diet, with a sex ratio of 1.06:1, 0.86:1, and 1:1 for tobacco, Chinese cabbage, and artificial diet, respectively.

### 3.2. Age-Specific Survivorship, Life Expectancy, and Fecundity

**Figure 1:**
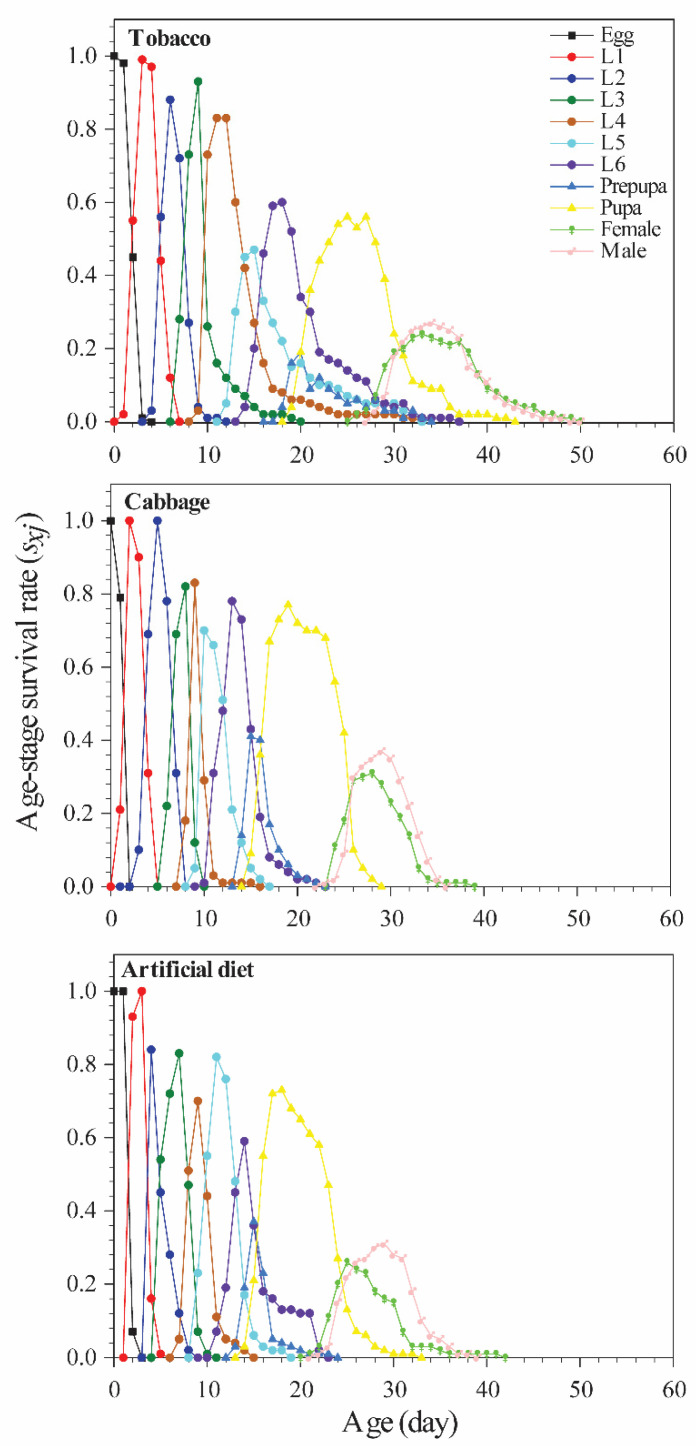
Age-stage specific survival rate (*s_xj_*) of *Spodoptera litura* reared on different diets.

**Figure 2:**
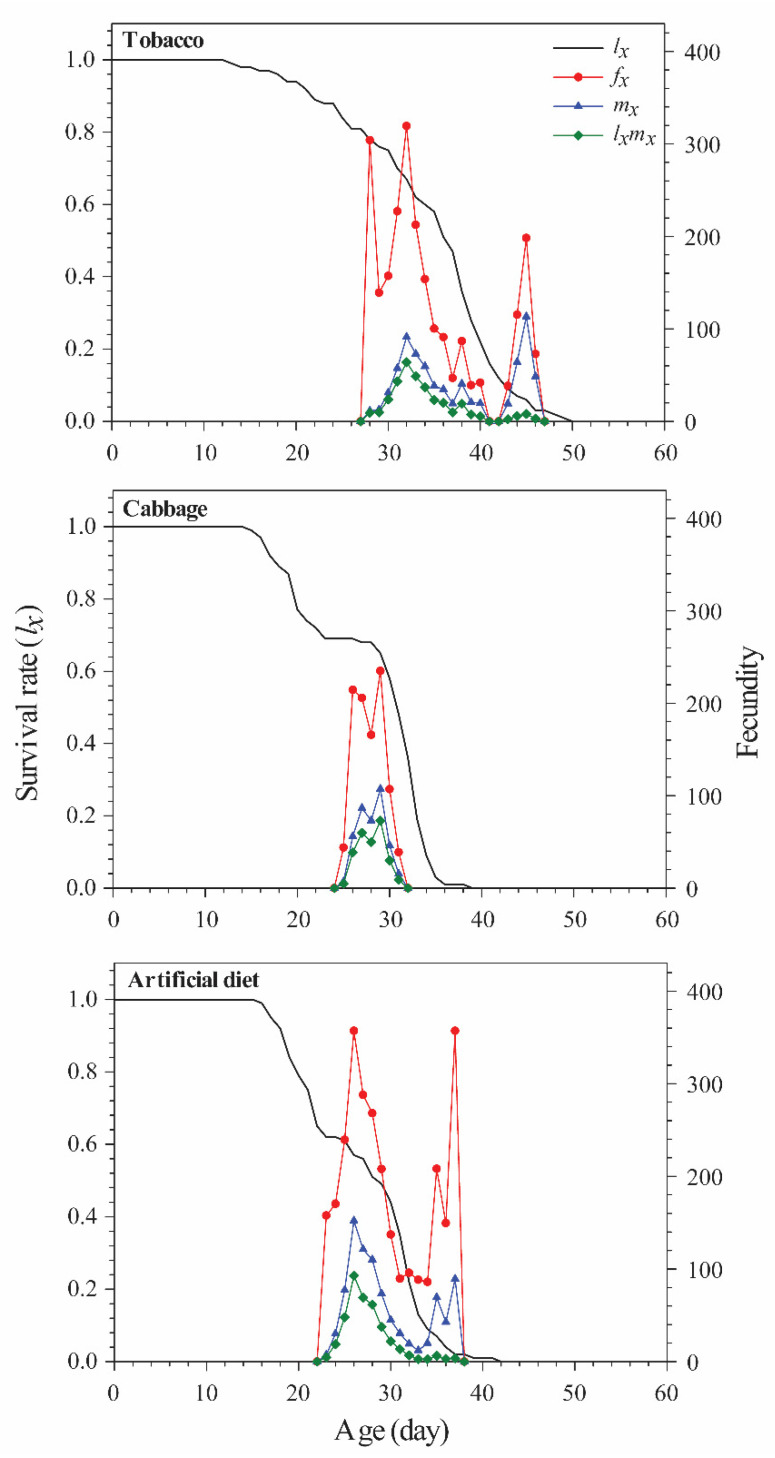
Age-specific survival rate (*l_x_*), female age-specific (*f_xj_*), age-specific fecundity of the total population (*m_x_*), and age-specific maternity (*l_x_m_x_*) of *Spodoptera litura* reared on different diets.

The age-stage specific survival rate (*s_xj_*) estimates the probability that an egg will survive to age *x* and stage *j* (Figure 1), and the age-specific survival rate (*l_x_*) is a simplified view of the survival rate of a whole cohort (Figure 2). In the present study, the survival rate was 67% for Chinese cabbage, 62% for tobacco, and 62% for the artificial diet; these groups showed decreased survival rates beginning at days 15, 13, and 16, respectively (and rapidly declining survivorship beginning at days 31, 36, and 31, respectively). The age-stage fecundity (*f_xj_*), age-specific fecundity (*m_x_*), and age-specific net maternity (*l_x_m_x_*) are shown in Figure 2. The values differed remarkably by diet. For example, adults fed tobacco, Chinese cabbage, or the artificial diet started reproducing at age 27 d, 24 d, and 22 d, respectively. The first peak of *m_x_* occurred on day 32 with 72.93 offspring in the tobacco group, day 28 with 107.09 offspring in the Chinese cabbage group, and day 25 with 152.16 offspring in the artificial diet group.

**Figure 3:**
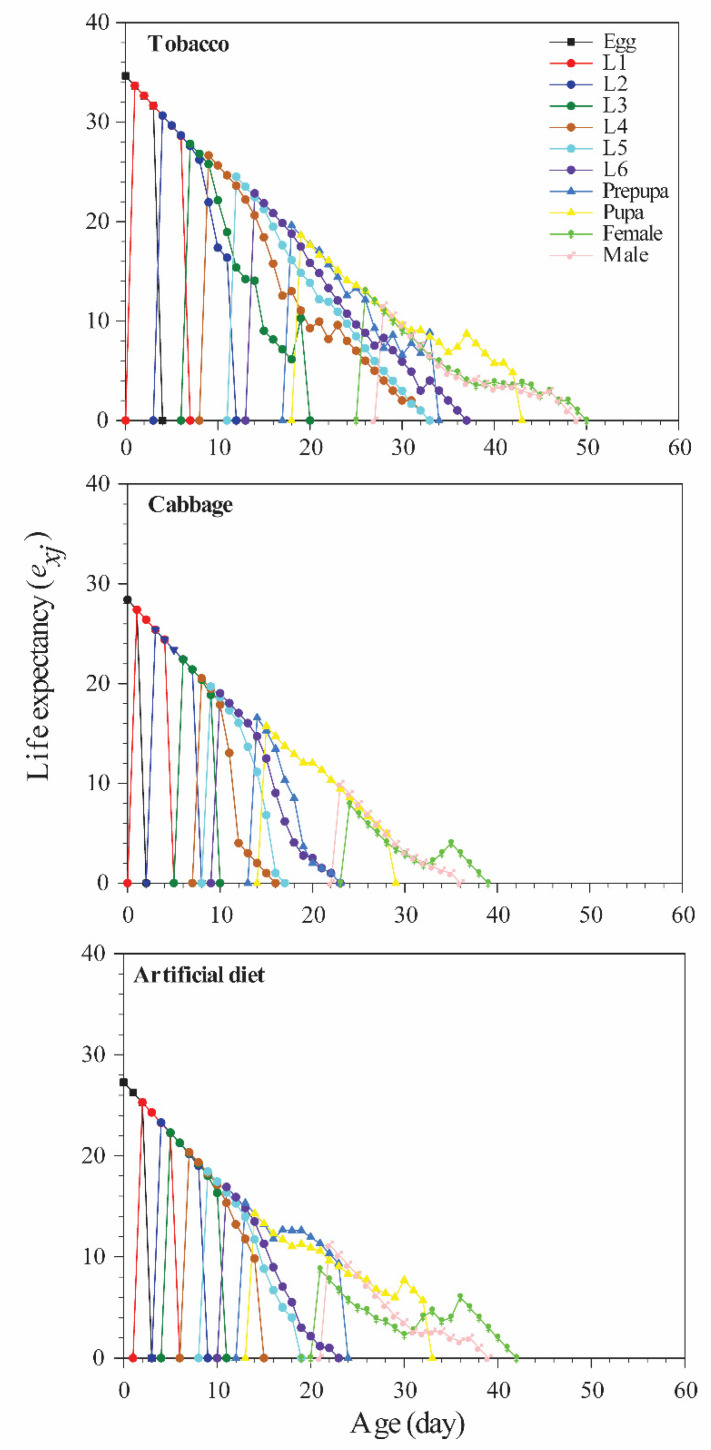
Age-specific life expectancy (*e_xj_*) of *Spodoptera litura* reared on different diets.

**Figure 4:**
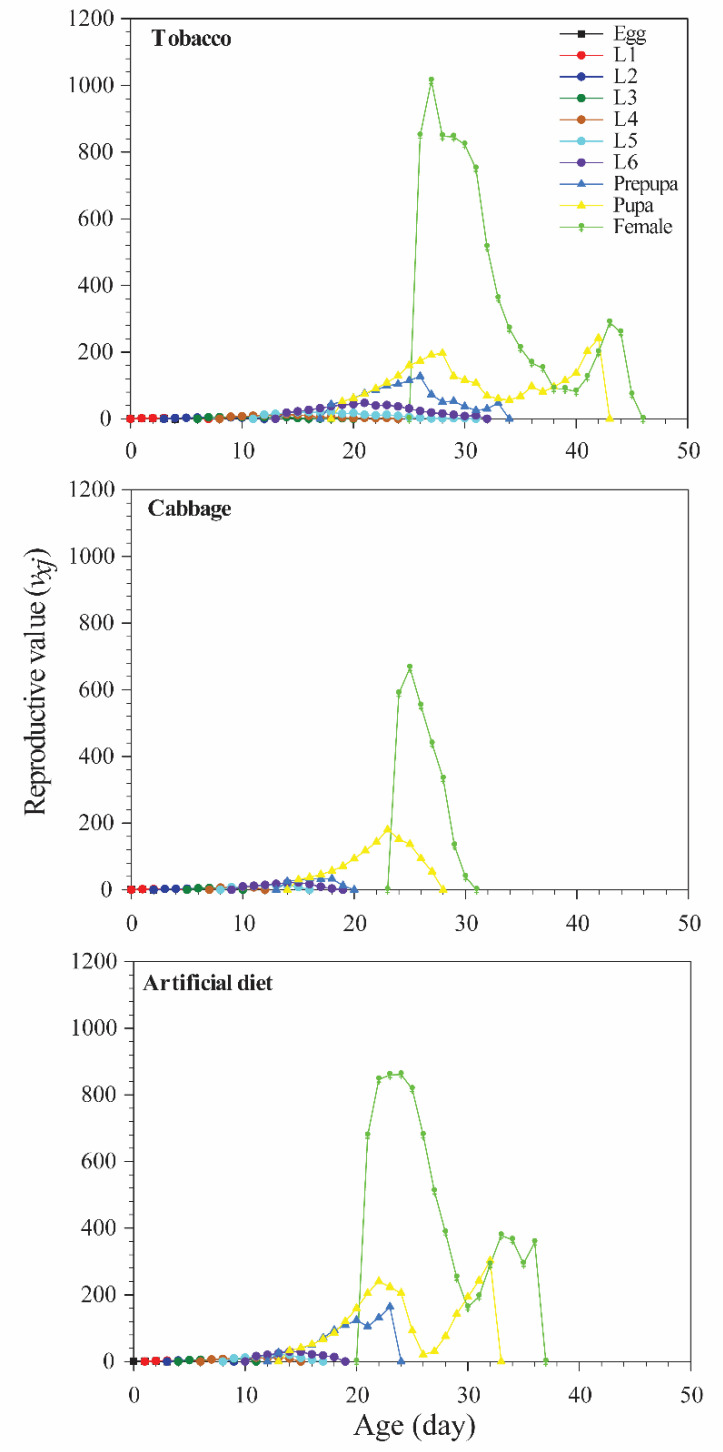
Age-stage-specific reproductive value (*v_xj_*) of *Spodoptera litura* reared on different diets.

The life expectancies of eggs, larvae, pupae, and adult stages were mostly higher in the tobacco group than in the other groups (Figure 3). Reproductive value (*v_xj_*) was used to assess the contribution of an individual of age *x* and stage *j* to the future population. Diet had a significant effect on oviposition period and fecundity (Table 1; Figure 4), with the highest *v_xj_* value (1013) at age 27 days in the tobacco group and the lowest value at age 25 days (666) in the cabbage group.

### 3.3. Life Table Parameters

Regarding population parameters, the intrinsic rate of increase (*r*) and the finite rate of increase (*λ*) were all significantly higher in the artificial group (Table 2), and the mean generation time (*T*) was obviously shorter.

### 3.4. Population Projections

**Figure 5:**
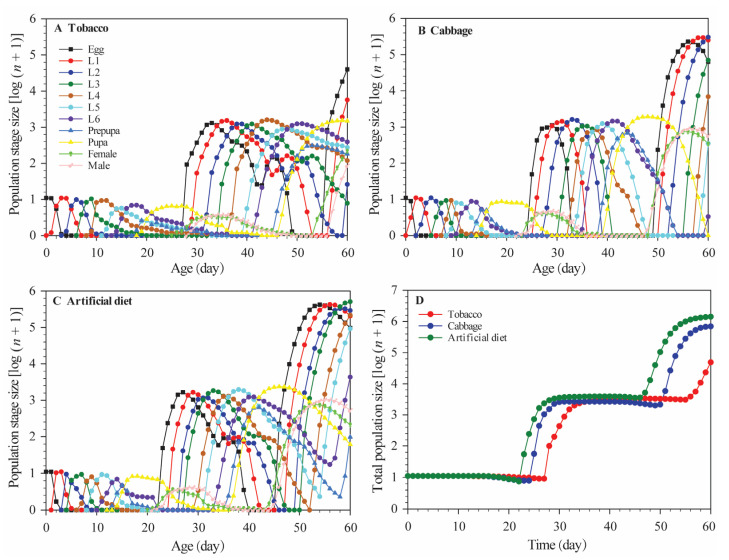
Population projections of *Spodoptera litura* in different stages fed (**A**) Chinese cabbage; (**B**) tobacco; (**C**) artificial diet; (**D**) represents the mean for the overall population of each diet group.

Figure 5 plots the simulated population growth of each diet group and by each developmental stage (and for each group overall). The estimates were generally lower for the tobacco group than the other two groups.

### 3.5. Mate Choice

**Figure 6:**
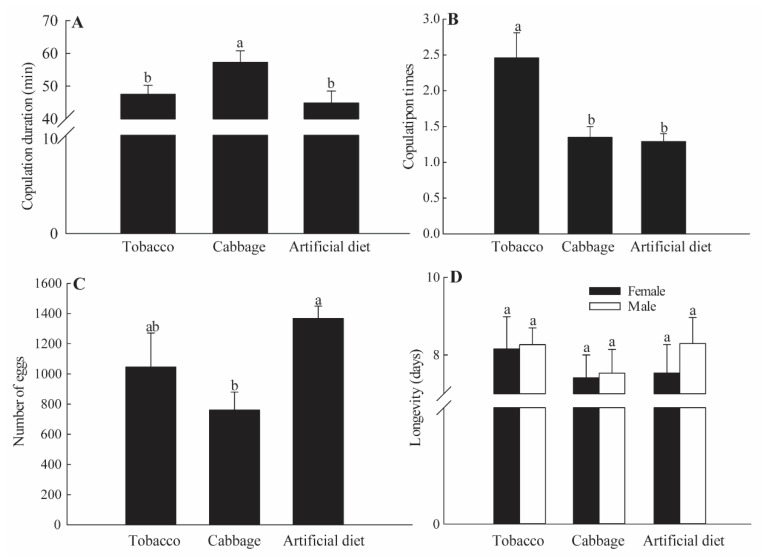
Copulation duration (**A**); copulation times (**B**); the number of eggs (**C**); and (**D**) longevity of *Spodoptera litura* in non-choice tests. Values are the mean ± SE (*n* = 30), with different lowercase letters indicating significant differences (*p* < 0.05, Tukey’s HSD multiple range test).

In non-choice tests, copulation events, copulation durations, and numbers of eggs significantly differed by group (Figure 6). The tobacco group showed the highest number of copulation events, with a higher probability of multiple matings, whereas the cabbage group had the highest copulation durations. However, the number of eggs was greatest (1368) in the artificial diet group.

Table 3 shows the results of the choice tests. We found that females significantly preferred males reared on the same diet they had consumed during development, especially in the artificial diet group in which 90% of females made that choice.

## 4. Discussion

The development and mating behavior of phytophagous insects is often linked to host plants in a variety of ways [37]. Host plant quality is a key factor in the survival and fecundity of insects [5,38]. The nutritional value and food quality of host plants or artificial diets are critical for the fitness of most insects [7]. In this study, we found that *S. litura* developed more quickly on the artificial diet than on tobacco or cabbage and also showed significantly higher fecundity. However, females fed artificial diets rarely mated with males fed natural diets (tobacco and Chinese cabbage).

Insect development is strongly influenced by diet [4,22,39], and the consumption of different plant species can cause differences in life history traits and survival [6,40,41,42]. Compared to larvae reared on natural hosts such as Chinese cabbage and tobacco, those reared on artificial diets tend to have shorter developmental times. The results of this study are consistent with previous study [9,10,24,43]. For instance, in Su et al. [44], *Neoseiulus bicaudus* (Wainstein) mites reared on an artificial diet developed more slowly than those reared on a natural diet of *Tyrophagus putrescentiae* (Schrank). Prolonged development time is associated with a lack of basic nutrients [45]. In most Lepidoptera species, this can also lead to a delay in the developmental period [39]. Artificial diets contain a variety of nutrients and micronutrients, including proteins, lipids, phosphorus, and other minerals, which play an important role in insect development [10,46,47,48,49]. Protein restriction in particular may adversely affect developmental time [50,51]. In general, artificial diets are more suitable for the development of Lepidoptera species than a single natural host [10,43]. Therefore, the mass rearing of insects generally involves an artificial diet [24,26,27].

Different nutrients may influence insect fecundity differently [52,53]. In our study, the artificial diet significantly enhanced the fecundity of *S. litura*. However, the longevity of female larvae fed an artificial diet is obviously decreased. Similarly, *Spodoptera eridania* (Stoll) [10] and *Bradysia odoriphaga* Yang and Zhang [43] show higher fecundity but lower longevity when reared on an artificial diet versus host plants. Hence, there seems to be a trade-off between fecundity and longevity in female adults [50]. This may be due to limited internal resources, in that an individual cannot invest in both development and reproduction at the same time [54,55]. In addition, we found that the fecundity of *S. litura* was significantly higher when reared on tobacco than when fed Chinese cabbage. Xue et al. [21] found a similar result, where *S. litura* oviposited more eggs on tobacco than those reared on cabbage. Wu et al. [56] reported that *S. litura* fed on tobacco have multiple matings compared to those fed on cabbage, and that this further enhances the number of eggs produced. *Spodoptera litura* fecundity is affected by several factors [22,25,57], among which the species and quality of the diet is an important one.

Host plant preferences during the larval stages of insects can lead to both phenotypic plasticity and behavioral isolation [3,58]. Our results indicate that the mating parameters of *S. litura* significantly differed by diet. Those which consumed tobacco mated on average 2.46 times, so they were more likely to have multiple matings, which is in line with Wu et al. [56]. *Sitophilus oryzae* (L.) feed on different hosts, and this leads to different mating durations, being significantly longer when reared on wheat than on barley [59]. We evaluated mate choices by females fed different diets, as mate discrimination plays a major role in sexual isolation [31]. We found that females preferred to mate with males fed the same diet they had consumed as larvae. Similar results have been found in mustard leaf beetles [14]. Our results indicate that mate choice in *S. litura* is a population characteristic that is dependent on diet. This might be attributable to different diets causing differences in activity rhythms. The sexual activities of many moth species show specific rhythms [60]. Indeed, this has been observed in *S. litura*, and in a previous study [56], its first mating peak occurred 2 h after dark in a tobacco group but 5 h after dark in a cabbage group. Such diet-induced differences in mating peak may affect mating choice. Moreover, diet affects courtship behavior by influencing the composition of epicuticular hydrocarbons [58]. Furthermore, artificial diets are rich in protein, and dietary protein strongly increases the expression of male morphological traits, which are direct targets of mating selection [50]. We suspect that this is the main reason why females fed artificial diet rarely mated with males fed tobacco and cabbage in our study.

The results for *S. litura* feeding on the artificial diet may not reflect the occurrence rule of insects in the field. Host plants directly affect the fitness of insects [4,57], and the fitness of a pest population reflects its potential to damage host plants [22]. Overall, our study suggests that different diets lead to significant differences in the development, fecundity, survival, and mating choices of *S. litura*. Populations fed an artificial diet have a short developmental time and oviposit many eggs—two characteristics that are hugely beneficial to the mass rearing of *S. litura*. However, we also found that females very strongly preferred to mate with males fed the same diet. Specifically, 90% of females fed the artificial diet mated only with males fed that diet. Hence, diet can also lead to premating to isolation in *S. litura*, and artificially raised moths will have different behavioral parameters than moths raised under field conditions. Nonetheless, an increasing number of research groups are mass rearing larvae on artificial diets and conducting experiments on the resulting populations [23,26,27]. Therefore, we should consider whether the parameters for populations fed an artificial diet reflects population dynamics in the field.

## 5. Conclusions

This research indicates that the life parameters and assortative mating of *S. litura* differ depending on its diet. Our results may provide a theoretical basis for the mass rearing of test insects. In addition, our study helps clarify assortative mating in this species. More attention should be paid to studying the differentiation of adaptive coordination and diet impact on the mating signals of *S. litura*. Further clarification is needed regarding the effect of diet on phenotypic plasticity and its role in assortative mating. When experimental insects are fed an artificial diet, researchers should bear in mind that their results may not reflect the equivalent outcomes they would observe when the insect diet is the host plants in the field.

## Figures and Tables

**Table 1:** Mean duration of life stages and fecundity of *Spodoptera litura* fed different diets.

Stage/Parameter	*n*	Tobacco	*n*	Cabbage	*n*	Artificial Diet
Female						
Egg	32	2.53 ± 0.11 a	32	1.78 ± 0.07 c	31	2.03 ± 0.03 b
L1	32	2.88 ± 0.16 a	32	2.34 ± 0.09 b	31	2.06 ± 0.04 c
L2	32	2.56 ± 0.16 b	32	3.06 ± 0.12 a	31	1.94 ± 0.18 c
L3	32	2.56 ± 0.18 a	32	1.75 ± 0.11 b	31	2.42 ± 0.16 a
L4	32	3.78 ± 0.28 a	32	1.28 ± 0.09 c	31	1.65 ± 0.12 b
L5	32	2.81 ± 0.23 a	32	2.16 ± 0.19 b	31	2.87 ± 0.12 a
L6	32	4.5 ± 0.19 a	32	3.19 ± 0.16 b	31	2.03 ± 0.17 c
Prepupa	32	1.28 ± 0.09 a	32	1.06 ± 0.04 b	31	1.23 ± 0.23 ab
Pupa	32	8.28 ± 0.24 ab	32	8.59 ± 0.11 a	31	8.13 ± 0.14 b
Preadult	32	31.28 ± 0.72 a	32	25.22 ± 0.19 b	31	24.35 ± 0.41 b
Female adult longevity (d)	32	8.59 ± 0.42 ab	32	6.69 ± 0.27 b	31	8.19 ± 0.27 a
APOP (d)	31	0.94 ± 0.06 a	31	0.9 ± 0.05 a	31	0.87 ± 0.06 a
TPOP (d)	31	31.94 ± 0.72 a	31	26.16 ± 0.21 b	31	25.23 ± 0.42 c
Female total longevity (d)	32	39.78 ± 0.73 a	32	31.91 ± 0.39 b	31	30.19 ± 0.62 c
Oviposition days	32	2.45 ± 0.13 a	31	2.06 ± 0.13 b	31	2.58 ± 0.11 a
Fecundity (eggs/female)	32	1058.69 ± 76.05 a	32	826.22 ± 72.72 b	31	1265.68 ± 75.99 a
Male						
Egg	30	2.45 ± 0.13 a	37	1.76 ± 0.07 c	31	2.1 ± 0.05 b
L1	30	3 ± 0.17 a	37	2.46 ± 0.08 b	31	2.1 ± 0.05 c
L2	30	2.3 ± 0.18 b	37	2.84 ± 0.11 a	31	1.52 ± 0.17 c
L3	30	2.5 ± 0.13 a	37	1.92 ± 0.12 b	31	2.77 ± 0.17 a
L4	30	3.63 ± 0.13 a	37	1.27 ± 0.07 c	31	1.87 ± 0.14 b
L5	30	2.4 ± 0.18 b	37	2.16 ± 0.18 b	31	2.94 ± 0.15 a
L6	30	4.1 ± 1.06 a	37	2.81 ± 0.13 b	31	1.9 ± 0.12 c
Prepupa	30	1.27 ± 0.1 ab	37	1.41 ± 0.08 a	31	1.1 ± 0.05 b
Pupa	30	9.8 ± 0.14 a	37	9.41 ± 0.1 b	31	8.71 ± 0.19 c
Preadult	30	30.83 ± 0.41 a	37	26.03 ± 0.19 b	30	25 ± 0.29 c
Male adult longevity (d)	30	8.83 ± 0.53 a	37	6.92 ± 0.18 b	30	8.19 ± 0.27 a
Male total longevity (d)	30	39.87 ± 0.6 a	37	32.46 ± 0.24 b	30	33.19 ± 0.38 b

Values are means ± SE. Different letters within a row indicate significant differences among treatments (*p* < 0.05). APOP: female pre-ovipositional period; TPOP: the total pre-ovipositional period. Standard errors were estimated by using 100,000 bootstrap resampling.

**Table 2:** Population parameters of *Spodoptera litura* fed different diets.

Parameter	Tobacco	Cabbage	Artificial Diet
*R* (d^−1^)	0.18 ± 0.006 c	0.20 ± 0.006 b	0.22 ± 0.007 a
*λ* (d^−1^)	1.19 ± 0.007 c	1.22 ± 0.008 b	1.24 ± 0.008 a
*R*_0_ (offspring per individual)	338.78 ± 55.10 a	264.39 ± 44.35 a	392.36 ± 62.74 a
*T* (d^−1^)	32.87 ± 0.48 a	27.83 ± 0.23 b	26.88 ± 0.32 c

Values are means ± SE. Means followed by different letters in same row are significantly different. (*p* < 0.05).

**Table 3:** Mate choices of females by diet.

Gender	Diet	Male		
		Cabbage	Tobacco	Artificial Diet
Female	Cabbage	0.60 ± 011 a	0.30 ± 0.11 ab	0.10 ± 0.07 b
	Tobacco	0.20 ± 0.09 b	0.55 ± 0.11 a	0.25 ± 0.10 ab
	Artificial Diet	0.60 ± 011 a	0.30 ± 0.11 ab	0.10 ± 0.07 b

Values are means ± SE. Means followed by different letters in same row are significantly different. (*p* < 0.05).

## Data Availability

Date available on request.
